# Molecular and morphological characterization of three new species of avian Onchocercidae (Nematoda) with emphasis on circulating microfilariae

**DOI:** 10.1186/s13071-021-04614-8

**Published:** 2021-03-05

**Authors:** Rasa Binkienė, Carolina Romeiro Fernandes Chagas, Rasa Bernotienė, Gediminas Valkiūnas

**Affiliations:** Nature Research Centre, Akademijos 2, Vilnius, Lithuania

**Keywords:** Avian blood parasites, Filarioidea nematodes, New species, Microfilaria, Morphology, Molecular characterization, *cox1*, *28S*

## Abstract

**Background:**

Blood parasites have been the subject of much research, with numerous reports of the presence of microfilariae in the peripheral blood (circulating microfilariae) of birds belonging to many orders. Current limitations in molecular characterization methods and species identification using morphological characters of circulating microfilariae are major obstacles to improving our understanding the biology of Filarioidea species, particularly in wildlife. The aim of this study was to partially fill these gaps, with particular emphasis on morphological features of microfilariae, which are the most readily accessible stages of these pathogens.

**Methods:**

Peripheral blood samples of 206 birds belonging to genera *Acrocephalus* (five species) and *Sylvia* (five species) were examined using the buffy coat method to process the blood samples for the presence of microfilariae. Positive birds were dissected to collect adult nematodes. Microfilariae and adult nematodes were described, and sequences of their mitochondrial cytochrome* c* oxidase subunit I and nuclear *28S* rDNA gene fragments were obtained and used for molecular characterization and Bayesian phylogenetic inferences.

**Results:**

Overall prevalence of microfilariae was 2.9%. Microfilariae were found in the blood samples from six birds (2 *Acrocephalus scirpaceus* and 1 each of *A. arundinaceus*, *Sylvia atricapilla*,* S. borin* and *S. curruca*), which were dissected. All parasite species observed were new. *Eufilaria acrocephalusi* sp. n. and *Eufilaria sylviae* sp. n. were present in subcutaneous, peritracheal and periesophageal connective tissues in *A. scirpaceus* and *S. borin,* respectively. *Splendidofilaria bartletti* sp. n. was found in finger joins of *S. atricapilla.* Illustrations of microfilariae and adult nematodes are shown, and morphological and phylogenetic analyses identified the DNA barcode haplotypes that are associated with these species. Phylogenetic analysis places the parasites of different genera in different closely related clades.

**Conclusions:**

Adult nematode morphological characters, which have been traditionally used in the taxonomy of Filarioidea species, have a phylogenetic value. Importantly, in our study parasites of different genera were readily distinguishable based on the morphology of their microfilariae. The link between molecular and morphology data requires more study in Filarioidea species research, particularly because this approach provides new knowledge on species identity using only readily accessible blood stages (microfilariae), thereby avoiding host dissection and thus minimizing harm to wildlife during research.
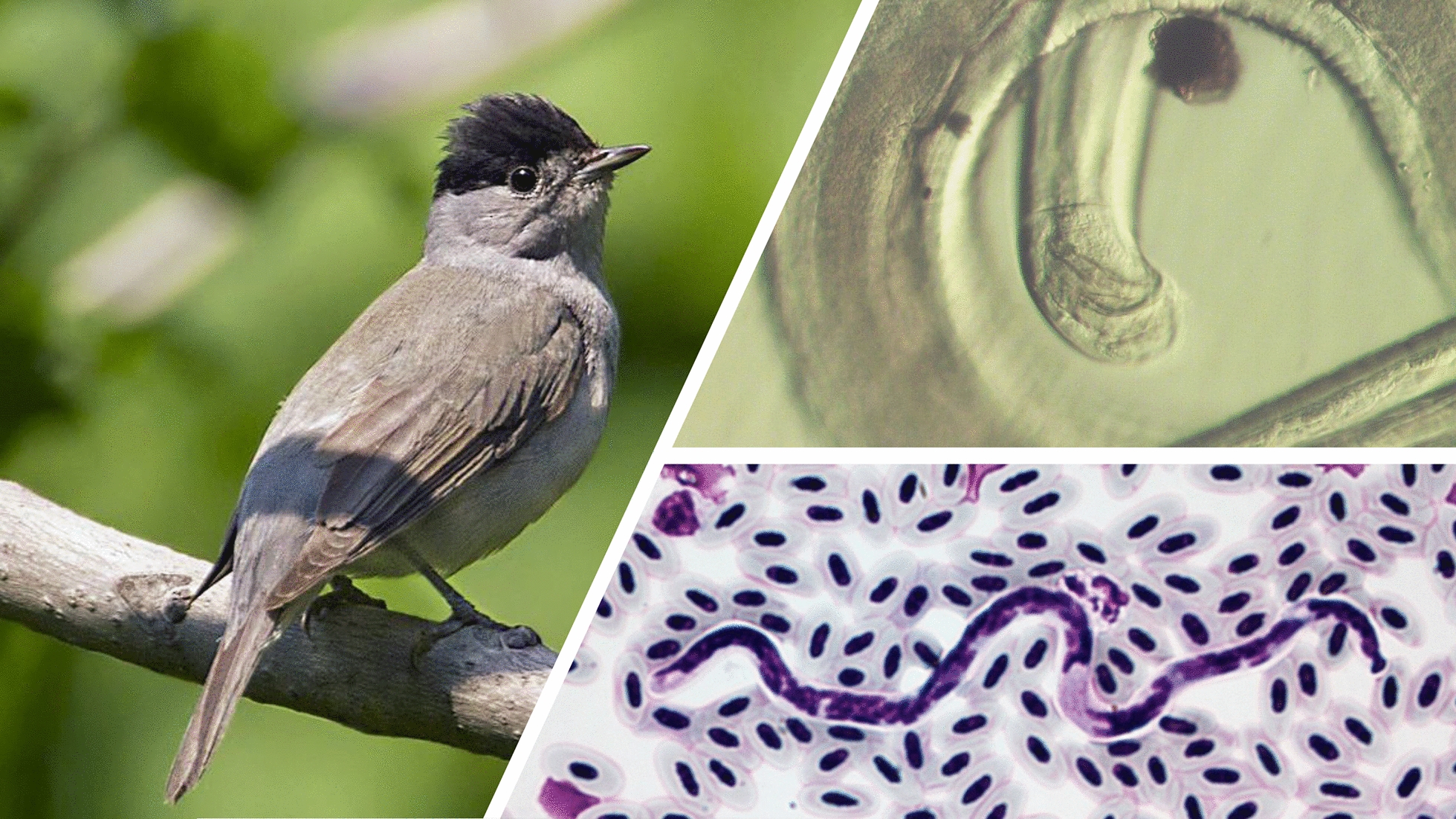

## Background

Blood parasites have been the subject of much research, with numerous reports of microfilariae of nematodes of the superfamily Filarioidea being present in the peripheral blood (circulating microfilariae) of birds belonging to many orders [1–3]. Filarioid nematodes are highly specialized parasites of tissues and tissue spaces of birds, mammals, amphibians and reptiles [4, 5]. Filarioids of birds belong to the family Onchocercidae (subfamilies Dirofilariinae, Onchocercinae, Splendidofilariinae and Lemdaniinae) and have a worldwide distribution [4, 5]. Adults worms occur in different tissues and cavities of avian hosts; therefore, it is challenging to determine their presence in the final host. Although there are many studies on the filarioids of birds, the majority of these have focused on economically important or pet bird species [4], and much less data are available on the parasites of birds naturally occurring in the wild state, including the widely distributed warbles belonging to genera *Sylvia* and *Acrocephalus*. The species diversity of avian filarioid nematodes remains insufficiently explored, with the last available description of a new species published by Bartlett in 1992 [6].

Mature females of filarioid nematodes produce microfilariae, which are released in the host body, enter the circulatory system and inhabit the blood or skin. Microfilariae of different nematode species are transmitted by various hematophagous arthropods, including biting midges, black flies, fleas, mosquitoes, lice, mites and ticks [5]. The microfilariae can be readily detected in the peripheral blood and have been reported in over 300 species of birds belonging to many orders [1–3]; however, there has been little improvement in our understanding of the biology of these helminths during the past 30 years. In general, microfilariae live longer than adults, so it is easier to detect them in the blood than to find adults worms in tissues [4]. Animals need to be euthanized to find the adult worms, which restricts sample collection in wildlife. The identification of parasite species using morphological characters of blood microfilaria is possible, but the methodology remains insufficiently developed due to the similarities in the morphology of microfilaria. Molecular-based methods could be useful as a means to simplify the detection of filarioids in final and intermediate hosts and species identification [7–9]. However, such methods have not yet been sufficiently developed in avian parasites. Despite the first molecular study on filarial parasites being published at the end of the last century [10], only a few studies have addressed challenge of the molecular characterization and phylogeny of filarioids using genetic material obtained from adult avian filarioid nematodes. Of the approximately 160 filarioid species described to date that parasitize birds, DNA sequences of only six species are currently available for the phylogenetic analysis of Onchocercidae nematodes: *Chandlerella quiscali* (von Linstow, 1904), *Splendidofilaria* sp., *Eulimdana clava* (Wedl, 1856), *Cardiofilaria pavlovskyi* Storm, 1937, *Aproctella alessandroi* Bain, Petit, Kosek and Chabaud, 1981, *Pelecitus fulicaeatrae* (Diesing, 1861) [11–13]. Consequently, due to this very limited DNA sequence data, molecular identification is usually impossible using microfilariae stages, which are often readily detectable and have been often seen in the blood of birds or in mosquitoes [1, 2, 14–19].

The insufficiently developed techniques for molecular characterization of avian Filarioidea species as well as the poorly developed methods for identifying parasites using morphological features of circulating microfilariae are major obstacles to improving our understanding the biology of these parasites, particularly in wildlife. The aim of this study was to contribute to filling this gap, with particular emphasis on morphological features of microfilariae, which are the most readily accessible stages of these pathogens in animals. We report here adults and microfilariae of three new proposed species of avian filarioid nematodes based on morphological and molecular characterization. Importantly, circulating microfilariae were identified to species levels and their DNA sequence information provides an opportunity to solely use blood samples for these nematode diagnostics in wildlife.

## Methods

### Material collection, fixation and staining

A total of 206 passeriform birds belonging to the genera *Acrocephalus* (five species) and *Sylvia* (five species) were caught at Ventės Ragas Ornithological Station, Lithuania (55°20′28.1″N, 21°11′25.3″E), during the spring migration in May 2018 (Table 1). The birds were captured with mist nets, zig-zag traps and large funnel type traps, following which they were ringed, identified and examined at the study site. Non-infected individuals were released after blood sampling (see description below). Infected birds were euthanized by decapitation and then dissected (see below).

**Table 1:** Prevalence of microfilariae circulating in peripheral blood in the examined adult bird species.

Species	No. of birds examined	No. of birds positive for microfilariae	No. of blood films examined	
			Total	Positive
*Acrocephalus arundinaceus*	16	1 (6.3%)^a^	7	3
* A. dumetorum*	2	0	–	–
* A. palustris*	42	0	–	–
* A. schoenobaenus*	51	0	–	–
* A. scirpaceus*	26	2 (7.7%)	10	7
*Sylvia atricapilla*	13	1 (7.7%)	8	6
* S. borin*	11	1 (9.1%)	14	0
* S. communis*	19	0	–	–
* S. curruca*	12	1 (8.3%)	3	0
* S. nisoria*	14	0	–	–

^a^ Infection prevalence is indicated in parentheses

Samples of blood (approx. 30 µl) were taken from the *vena ulnaris cutanea* (wing vein). A few drops of fresh blood were used to prepare three thin blood films for microscopic examination; the remaining blood (approximately 25 µl) was used in the buffy coat method to detect individuals infected with microfilariae [20]. More specifically, the heparinized capillary tubes with blood were centrifugated in a microhematocrit centrifuge for 5 min at 10,000 rpm, following each tube was placed above a glass slide and the buffy coat area was examined under low magnification. Only microfilariae-positive birds were used in this study. An additional 30–50 μl (approximately) of blood was taken from these birds, and several extra blood films were prepared for collection purposes; the remaining blood (approx. 20–30 µl) was fixed in 95% ethanol for molecular diagnosis.

All blood films were rapidly air-dried using a battery-powered fan, fixed in absolute methanol and stained with Giemsa [21]. They were examined at low magnification (200×). If microfilariae were present, the parasites were studied at medium (500×) and high (1000×) magnifications, and their images were prepared. Measurements were carried out using the DLTCam Viewer 3.7.8301 software (Delta Optical, Mińsk Mazowiecki, Poland). In microfilariae, total body length, headspace, tail length, maximum width of body and distance of fixed points from anterior extremity were measured (Table 2). Fixed point values were expressed as percentages of the total body length.

**Table 2:** Morphometric parameters and shape of microfilariae in the peripheral blood and liver of birds.

Character	Parasite species			
	*Eufilaria acrocephalusi*	*Eufilaria sylviae*	*Splendidofilaria bartletti*	
	Blood (*n* = 21)	Liver (*n* = 10)	Blood (*n* = 21)	Liver (*n* = 10)
Body size (μm)				
Length	146–184 (168 ± 10.5)	84–154 (121 ± 25)	150–174 (163 ± 6.7)	97–127 (113 ± 8.5)
Width	2.3–3.2 (2.9 ± 0.2)	3.7–5 (4.2 ± 0.7)	4.2–5.4 (4.9 ± 0.4)	3.5–5 (4.2 ± 0.4)
Cephalic space length	0–1.8 (1.1 ± 0.5)	2.5–5.8 (3.7 ± 1)	1.1–4.1 (2.2 ± 0.6)	1.3–2.3 (1.9 ± 0.5)
Inner body length	3–7 (5 ± 1)	2–8 (4 ± 1.8)	8–15 (11± 1.9)	7–12 (9 ± 1.6)
Distances^a^				
Nerve ring	34–41 (37 ± 2.1)	20–32 (2 6 ± 3.6)	31–39 (35 ± 2.4)	31–34 (27 ± 2.2)
	[20–25 (22 ± 1.2)]	[17–25 (22 ± 2.7)]	[19–24 (22 ± 1.4)]	[21–25 (24 ±1.2)]
Excretory pore	49–64 (57 ± 4.6)	28–48 (36 ± 7.1)	55–64 (59 ± 2.7)	34–49 (41 ± 5)
	[32–38 (34 ± 1.9)]	[30–34 (32 ± 1.6)]	[34–39 (36 ± 1.1)]	[32–41 (37 ± 3.3)]
Inner body anterior end	81–99 (90 ± 5.2)	48–89 (67 ± 14)	84–100 (93 ± 4.3)	55–75 (65 ± 5.6)
	[52–56 (54 ± 1.2)]	[54–58 (56 ± 1.1)]	[56–60 (57 ± 1.2)]	[55–59 (57 ± 1.3)]
Inner body posterior end	85–104 (95 ± 3.4)	50–94 (71 ± 16)	94–115 (105 ± 5.1)	63–73 (73 ± 6.7)
	[55–60 (57 ± 0.1.4)]	[58–61 (60 ± 1)]	[62–66 (64 ± 1.2)]	[62–69 (65 ± 2.3)]
Anal pore	121–54 (139 ± 9.4)	68–125 (94 ± 21.5)	130–156 (143 ± 6.5)	83–114 (99 ± 8.7)
	[81–87 (83 ± 1.6)]	[79–84 (81 ± 1.4)]	[86–90 (88 ± 1.3)]	[86–90 (87 ± 1.4)]
Shape				
Anterior extremity	Rounded	Rounded	Rounded	Rounded
Tale	Sharply pointed	Sharply pointed	Broadly rounded	Broadly rounded
Sheath	Absent	Absent	Present	Present

All measurements (with the exception of Shape) are given as the minimum and maximum values, followed in parenthesis by the arithmetic mean and standard deviation (SD). For Distances, the relative distance (in percentage) is also given, in brackets

^a^Distance from anterior extremity to the mentioned point is given

Individual birds infected with microfilariae were euthanized and immediately dissected. Dissected organs and tissues were placed in Petri dishes containing 0.9% saline solution, and the parasites were recovered using dissecting needles and pipettes under a stereomicroscope (model MБC-9, Lomo, Russia). The subcutaneous tissues, joints of leg and wings, brain, eyes, heart, connective tissues of trachea and esophagus, lungs, air sacs and body cavities were examined for adult filarioid nematodes. Wings and legs were separated from the body in the region of the humerus and femur, and joints were broken open in saline solution using dissecting needles. Inner organs, connective tissues and skin were compressed between two glasses and examined. Lungs, liver, spleen and kidneys were also examined for the presence of microfilaria: a drop of a mixture of saline solution with blood from the organs was placed on the objective glass slide, covered with a coverslip and examined under the microscope. Adult alive nematodes were examined under the microscope in 0.9% saline solution and then stored in 70% ethanol. For examination by light microscopy, the nematodes were cleared with glycerine [22]. Smears were also prepared from the blood collected from internal organs; these blood samples were fixed, stained and examined as blood films (see description above). All blood films were examined using an Olympus BX 51 light microscope (Olympus Corp., Tokyo, Japan) equipped with differential interference contrast optics and a digital image analysis system (Delta Optical DLTCam Viewer 3.7.8301).

Parasite species were identified using keys and original descriptions [6, 23–30]. Specimens used in the description and DNA sequences were deposited in the Natural History Museum of Geneva (MHNG) and in the Nature Research Centre (NRCL) in Lithuania (see Results, parasite description and accession numbers).

### DNA extraction, PCR and sequencing

Parasite DNA was extracted from blood, microfilaria-infected bird tissues and adult nematodes according to Stunžėnas et al. [31], with a minor modification according to Petkevičiūtė et al. [32]. In general, a sample was allowed to dry for 15–30 min on a sterile microscope glass slide before DNA extraction. Partial sequences of mitochondrial cytochrome* c* oxidase I (*cox1*) and nuclear *28S* rDNA (*28S*) genes were obtained and used in this study. During amplification of both gene sequences, the PCR mix was the same, consisting of a total volume of 25 μl and containing 13 μl of DreamTaq PCR Master Mix (Thermo Fisher Scientific, Vilnius, Lithuania), 8 μl of nuclease-free water, 1 μl of each primer and 2 μl of DNA template. A fragment of approximately 650 bp of *cox1* (without primers) was amplified using the primers COIintF (5′-TGATTGGTGGTTTTGGTAA-3′) and COIintR (5′-ATAAGTACGAGTATCAATATC-3′) [33, 34]. The touchdown cycling profile consisted of denaturation at 94 °C for 2 min; then 94 °C/45 s, 51 °C/45 s (reduced by 1 °C for every 2 cycles), 72 °C/1.5 min for 8 cycles; following by 94 °C/45 s, 45 °C/ 45 s, 72 °C/1.5 min for 25 cycles; and a final extension at 72 °C for 7 min. A fragment of approximately 765 bp (without primers) of *28S* was amplified using the primers Nematode 1 (5′-GCGGAGGAAAAGAAACTAA-3′) and Nematode 2 (5′-ATCCGTGTTTCAAGACGGG-3′) [14]. The cycling profile consisted of denaturation at 94 °C for 3 min; then 94 °C/30 s, 55 °C/30 s, 72 °C/45 s for 36 cycles; and a final extension at 72 °C for 7 min. The products of the PCRs were evaluated by electrophoresis in 1% agarose gel. A 2-μl sample of each PCR product was used to test the success of amplification for each performed action. Positive PCR products were sequenced from both the 5′- and 3′-ends with the same primers using the Big Dye Terminator V3.1 Cycle Sequencing kit and ABI PRISM™ 3100 capillary sequencing robot (Applied Biosystems, Foster City, CA, USA). Sequences were examined, edited and aligned using the BioEdit software in order to create a consensus sequence [35]. To confirm that adult filarioid nematodes and detected microfilariae belonged to the same species, the DNA sequences obtained from the adult worms and microfilariae-infected samples from the same bird individuals were compared. All sequences obtained in this study were deposited in GenBank (see Results, parasite description).

### Phylogenetic analysis

Consensus sequences obtained of the both genes (*cox1* and *28S*) were aligned with other sequences obtained from GenBank using MAFFT software version 7.470 [36] using the method L-INS-is (command: *mafft --thread 4 --threadtb 5 --threadit 0 --reorder --auto input > output*). Bayesian phylogenetic analyses was performed for sequences of each gene separately using MrBayes v3.2.0 software [37]. Best-fit evolution general reversible time was selected using MrModeltest2 software [38]. The analysis was conducted by analyzing 3 million generations, with the sample frequency set at every 100 generations. The first 25% of trees were discarded as a burn in. The consensus tree was visualized in FigTree v1.4.3 software [39]. A total of 30 sequences (8 from this study) and 29 sequences (10 from this study) were used in phylogenetic analysis of the *cox1* and *28S* genes, respectively. Pairwise distance (p-distance) was calculated between different sequences used in the phylogenetic analysis for each gene. This analysis was implemented in MEGAX software using the p-distance model with uniform rates of nucleotide substitutions (see Additional file 1: Table S1; Additional file 2: Table S2) [40].

## Results

A total of 137 adult birds of the genus *Acrocephalus* and 69 adult birds of the genus *Sylvia* were examined for the presence of circulating microfilariae (Table 1). Microfilariae were seen in six birds (2 common reed warblers *Acrocephalus scirpaceus*, 1 great reed warbler *Acrocephalus arundinaceus*, 1 Eurasian blackcap *Sylvia atricapilla,* one garden warbler *Sylvia borin* and one lesser whitethroat *Sylvia curruca*). The overall infection prevalence was 2.9%.

Adults of *Eufilaria* species were present in subcutaneous, peritracheal and perioesophageal connective tissues of the common reed warbler and garden warbler. *Splendidofilaria* species was found in the finger joins of the Eurasian blackcap*.* No adult worms of Onchocercidae species were detected in the great reed warbler and in the lesser whitethroat, but microfilariae were detected in the blood of these individuals. Morphology of the nematodes found did not match with that of any known species of Onchocercidae. Consequently, we designated these as new species and present their description in the following sections.

### Description of new nematode species of the family Onchocercidae

**Fig. 1:**
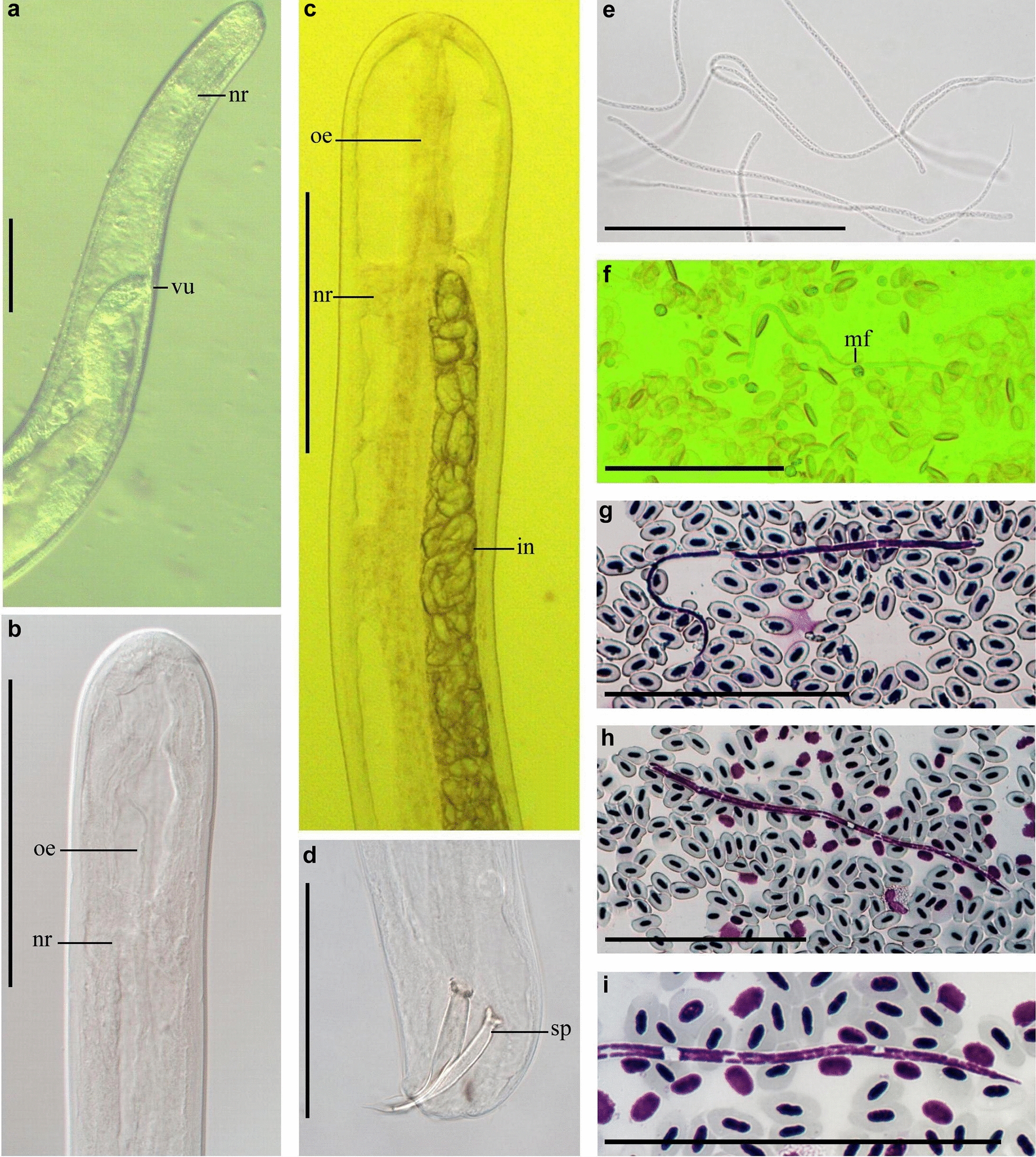
*Eufilaria* a*crocephalusi* sp. n. from *Acrocephalus scirpaceus* (common reed warbler). **a** Female anterior extremity (live worm). **b** Male anterior extremity (ethanol-fixed). **c** Female anterior extremity (ethanol-fixed). **d** Male spicules (ethanol-fixed). **e** Microfilariae obtained from uterus (female was ethanol-fixed). **f** Live microfilaria in peripheral blood. **g**, **h** Microfilariae from peripheral blood (methanol-fixed, Giemsa-stained blood films). **i** Posterior part of microfilaria from peripheral blood (Giemsa-stained). Note that all microfilariae have a thread-like appearance (**e**–**h**). Width is a distinctive feature of this parasite. All scale bars: 100 μm.* ib* inner body,* in* intestine,* mf* microfilariae,* nr* nerve ring,* oe* esophagus,* ov* ovary,* sp* spicula,* ut* uterus,* vu* vulva

**Fig. 2:**
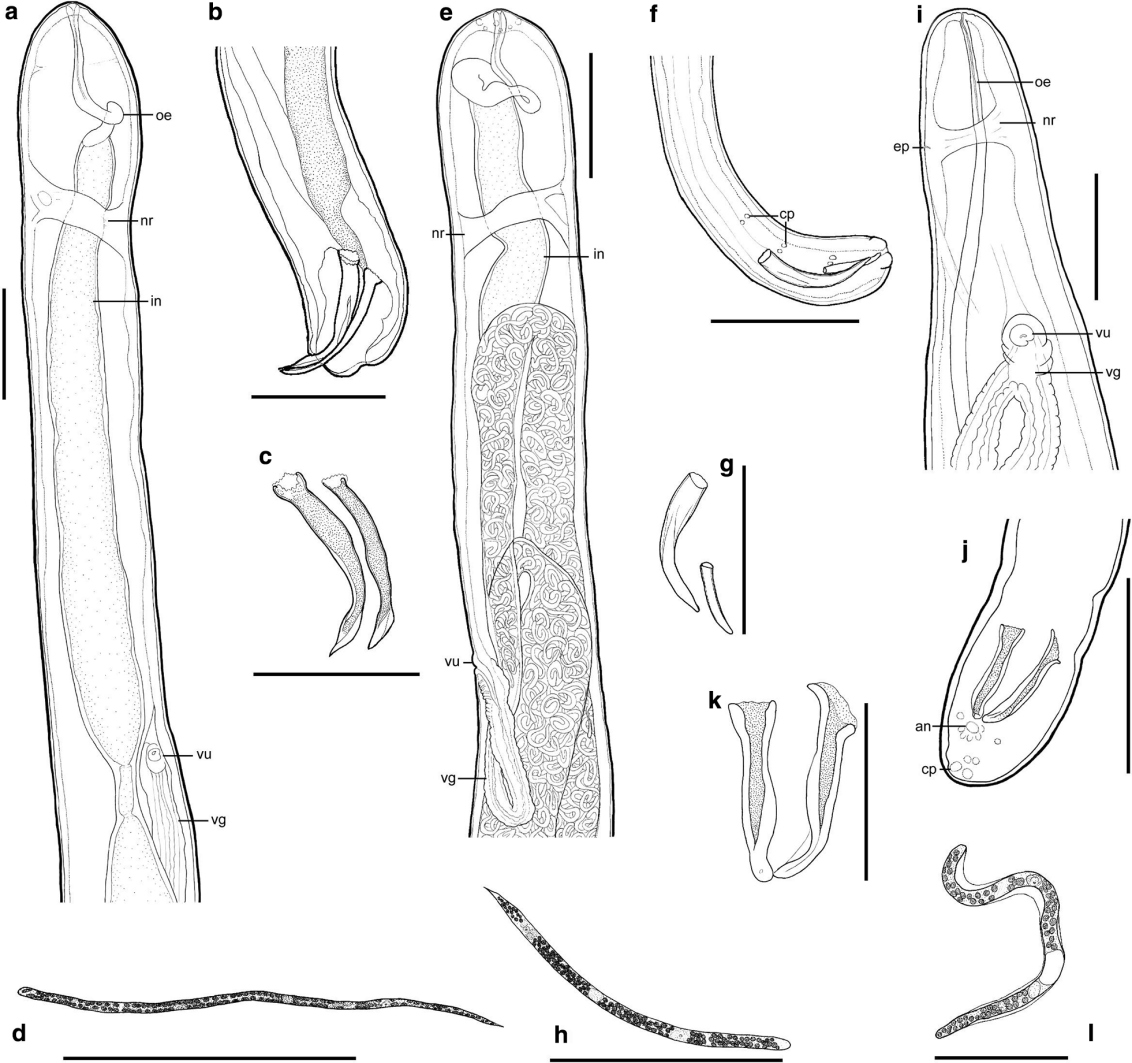
**a**–**d** *Eufilaria acrocephalusi* sp. n.. **a** Female anterior extremity, **b** male posterior extremity, **c** spicules, **d** microfilaria. **e**–**h** *Eufilaria sylviae* sp. n.. female anterior extremity (**e**), male posterior extremity (**g**), spicules (**g**), microfilaria (**h**). **i**–**l** *Splendidofilaria bartletti* sp. n.. female anterior extremity (**i**), male posterior extremity (**j**), spicules (**k**), microfilaria (**l**). Scale bars: **a**–**j** 100 μm; **k**, **l** 50 μm.* an* Anus,* cp* caudal papilla,* ep* excretory pore,* vg* vagina; see caption to Fig. 1 for other abbreviations

***Eufilaria acrocephalusi*** Binkienė sp. n. (Figs. 1, 2a–d)

***Description:*** Slender nematodes with bluntly rounded extremities (Fig. 1a-d). Male shorter than female (Table 3). Cuticle thin, smooth. Cephalic extremity with tiny and difficult to discern submedian papillae (Fig. 2a). Amphids not salient. Buccal capsule absent. Oral opening small. Excretory pore not seen. Pre-esophageal ring absent. Esophagus slender, not externally divided into muscular and glandular part (Figs. 1b, c, 2a). Junction of esophagus and intestines indistinct. Vulva does not extend the body surface, slightly concave (Figs. 1a, 2a). Vagina directed posteriorly from vulva, not convoluted (Fig. 2a). Uterus didelphic and opisthodelphic. Ovary bends at 275 μm from posterior extremity. Spicules slightly dissimilar, head demarcated from blade by constriction (Figs. 1d, 2b, c). Two pairs of adanal papillae are present. Posterior extremity of body is bluntly rounded. Anus of male subterminal (Fig. 2b). Microfilariae short (Table 2), without sheath, anterior extremity rounded (Figs. 1e-g, 2d), tail sharply pointed (Fig. 1i). Posterior nucleus oval and present at posterior extremity.

**Table 3:** Morphometric parameters of adult nematodes of new species found in birds of genus *Acrocephalus* and genus *Sylvia*

Characters	Parasite species					
	*Eufilaria acrocephalusi*		*Eufilaria sylviae*		*Splendidofilaria bartletti*	
	Male	Female	Male	Female	Male	Female
Body size						
Total length (mm)	13.7	26	3.7	10.1	3.5	11.4
Maximum width	80	187	52	129	122	261
Width at nerve ring	58	99	34	98	59	86
Width at vulva	–	114	–	105	–	129
Width at anus	59	49	41	61	55	115
Organ length						
Esophagus	171	193	194	225	364	–
Vagina	–	296	–	193	–	48
Left spicules	78	–	81	–	55	–
Right spicules	72	–	53	–	50	–
Distances						
Nerve ring^a^	138	171	94	154	75	80
Vulva^a^	–	671	–	526	–	267
Anus^b^	11	50	Subterminal	subterminal	36	151

All measurements are given in micrometers (μm), unless indicated otherwise. Measurements of each species were made using single specimens of different gender.

^a^Distance from anterior extremity to the mentioned point is given

^b^Distance from posterior extremity to the mentioned point is given

***Type host***: *Acrocephalus scirpaceus* (Hermann, 1804) (Passeriformes, Acrocephalidae).

***Location in host***: Adults in subcutaneous, peritracheal and perioesophageal connective tissues. Microfilariae seen in peripheral blood, capillaries of liver, lung and spleen.

***Type locality***: Ventės Ragas Ornithological Station (55°20′28.1″N, 21°11′25.3″E), Lithuania.

***Type specimens***: Holotype (female MHNG-INVE-137370) allotype (male MHNG-INVE-137371) were deposited in MHNG, voucher (EKOI HELMI 1027 female) used in DNA sequence was deposited in NRCL.

***ZooBank accesion***: The Life Science Identifier (LSID) for *Eufilaria acrocephalusi* sp. n. is urn:lsid:zoobank.org:act:A140A6A5-9FC2-42B5-8ECB-B39A2AD975E7.

***DNA sequences***: Sequences were obtained from two individual *Acrocephalus scirpaceus* hosts. In the first bird the mitochondrial *cox1* (GenBank accession number MT800766) and nuclear *28S* (MT802308) were generated from microfilaria; nuclear *28S* (MT802309) was generated from the adult worm. In the second bird, mitochondrial *cox1* (MT800768) and *28S* (MT802314) were generated from microfilaria; mitochondrial *cox1* (MT800769) and nuclear *28S* (MT802315) were generated from the adult worm.

***Etymology***: The species name refers to the genus name of the definitive host.

***Remarks***

The new species is most similar to and should be distinguished from *Eufilaria delicate* Supperer, 1958, *Eufilaria alii* (Deshmukh, 1968) and *Eufilaria buckleyi* Deshmukh, 1968 [27, 30].

*Eufilaria delicate* was described from the common blackbird *Turdus merula*, mistle thrush *Turdus viscivorus* and Eurasian jay *Garrulus glandarius* in Europe [29, 30, 41, 42]. This nematode has shorter spicules (58–65 μm and 50–61 μm) and thicker microfilariae (4–5 μm wide) than *E. acrocephalusi* sp. n. (Table 2).

*Eufilaria buckleyi* was described from the jungle bush quail *Perdicula asiatica* in India [27]. The vagina of this parasite is directed posteriorly in the beginning, and then turns anteriorly; female gonads reach the nerve ring. However, the vagina of *E. acrocephalusi* sp. n. is directed posteriorly and female gonads are postvaginal (Fig. 2a). The left spicules of both *E. acrocephalusi* sp. n. and *E. buckleyi* are similar in length (Table 3), but the right spicule of *E. buckleyi* is shorter (50–60 μm). Microfilariae of *E. buckleyi* are shorter 75–93 μm and slightly wider (3–4 μm) than those in *E. acrocephalusi* sp. n.

*Eufilaria alii* was described from the yellow-legged buttonquail *Turnix tanki* from India [27]. The male of *E. alii* is slightly smaller (8.03–11.7 mm), but wider (110–130 μm). The tail of the female is longer (80–160 μm), and the ovary extends behind the anus until the posterior extremity, while the tail of *E. acrocephalusi* sp. n. is short (58 μm) and the ovary ends before the anus. Microfilariae of *E. alii* are shorter 88–97 μm and wider (4–5 μm).

**Fig. 3:**
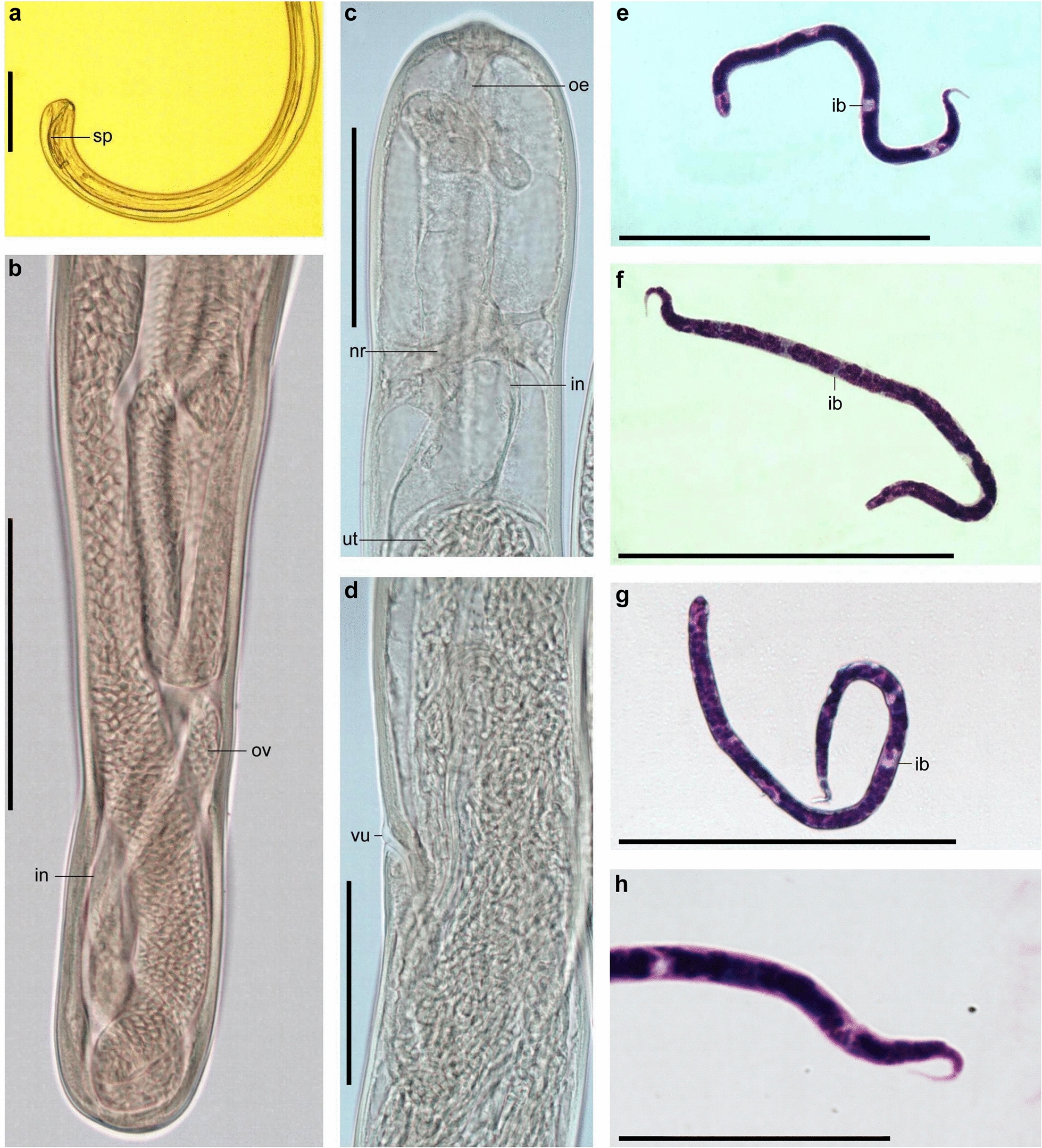
*Eufilaria sylviae* sp*.* n. from *Sylvia borin* (garden warbler). **a** Male posterior extremity (alive worm). **b** Female posterior extremity (ethanol-fixed). **c** Female anterior extremity (ethanol-fixed). **d** Vulva (ethanol-fixed). **e**–**g** Microfilariae from liver blood films (Giemsa-stained). **h** Posterior part of microfilaria from liver blood (Giemsa-stained). Note that all microfilariae have a sharply pointed, curved tail and small inner body. Scale bars: **a**–**g** 100 μm, **h** 30 μm. See captions to Figs. 1 and 2 for abbreviations

***Eufilaria sylviae*** Binkienė sp. n. (Figs. 2e–h, 3)

***Description:*** Slender nematodes with bluntly rounded extremities (Fig. 3a–c). Male shorter than female (Table 3). Cuticle thin, smooth. Buccal capsule absent. Oral opening small (Fig. 2e). Excretory pore not seen. Pre-esophageal ring absent. Esophagus slender, poorly developed (Figs. 2e, 3c). Junction of esophagus and intestines not clearly distinct. Vulva slightly extends the body surface (Figs. 2e, 3d). Vagina directed posteriorly from vulva, then turned anteriorly; female gonads almost reach the nerve ring, 236 μm from apex they turn backwards (Fig 2e). Uterus didelphic and opisthodelphic. Ovary up to posterior extremity (Fig. 3b). Spicules dissimilar in length (Fig. 2f, g). Three pairs of preanal (Fig. 2f) and two pairs adanal papillae present. Anus subterminal. Microfilaria short with transversely striated cuticle, without sheath (Figs. 2h, 3e–g). Anterior extremity rounded, tail sharply pointed (Fig. 3h), usually the part of tail without nucleus is curved (Table 2).

***Type host:*** *Sylvia borin* (Boddaert, 1783) (Passeriformes, Sylviidae).

***Location in host***: Adults in subcutaneous, peritracheal and perioesophageal connective tissues. Microfilariae in peripheral blood, capillaries of liver and lung.

***Type locality***: Ventės Ragas Ornithological Station (55°20′28.1″N, 21°11′25.3″E), Lithuanian Republic.

***Type specimens***: Holotype (female MHNG-INVE-137372) were deposited in MHNG.

***ZooBank accesion***: The Life Science Identifier (LSID) for *Eufilaria sylviae* sp. n. is urn:lsid:zoobank.org:act:6F832F4D-9415-4BB5-9A58-3F2439EEF7DF

***DNA sequences***: Mitochondrial *cox1* (GenBank accession number MT800770) and nuclear *28S* (MT802312) generated from microfilaria. Mitochondrial *cox1* (MT800771) and nuclear *28S* (MT802311) generated from adult worm.

***Etymology***: The species name refers to the genus name of the definitive host.

***Remarks***

The new species can be readily distinguished from other *Eufilaria* species by its body length, spicules length and position of female gonads.

Due to the body length, *E. sylviae* sp. n. is the most similar to *Eufilaria sergenti* Seurat, 1921 (male 3.3 mm, female 14 mm) that was found in the Spanish sparrow *Passer hispaniolensis* and white-crested laughingthrush *Garrulax leucolophus* in Spain [29]. However, the left spicule of *E. sergenti* is shorter (72 μm), the vulva is closer to the anterior extremity (250 μm from the apex) and the vagina is more than ten times longer (720 μm).

Due to the length of spicules and position of vulva, *E. sylviae* sp. n. is most similar to *Eufilaria buckleyi* (Deshmukh, 1968), the parasite of jungle bush quail *Perdicula asiatica* from India [27]. Different from *E. sylviae* sp. n.,* E. buckleyi* is longer (male 7.6–9 μm, female 25–34 μm), and the female tail is also longer (190–270 μm). Additionally, the first pair of male’s caudal papillae of *E. buckleyi* is located in parallel to the capitulum of spicules, while the first pair of the caudal papillae of *E. sylviae* sp. n. is located in front of the spicules.

**Fig. 4:**
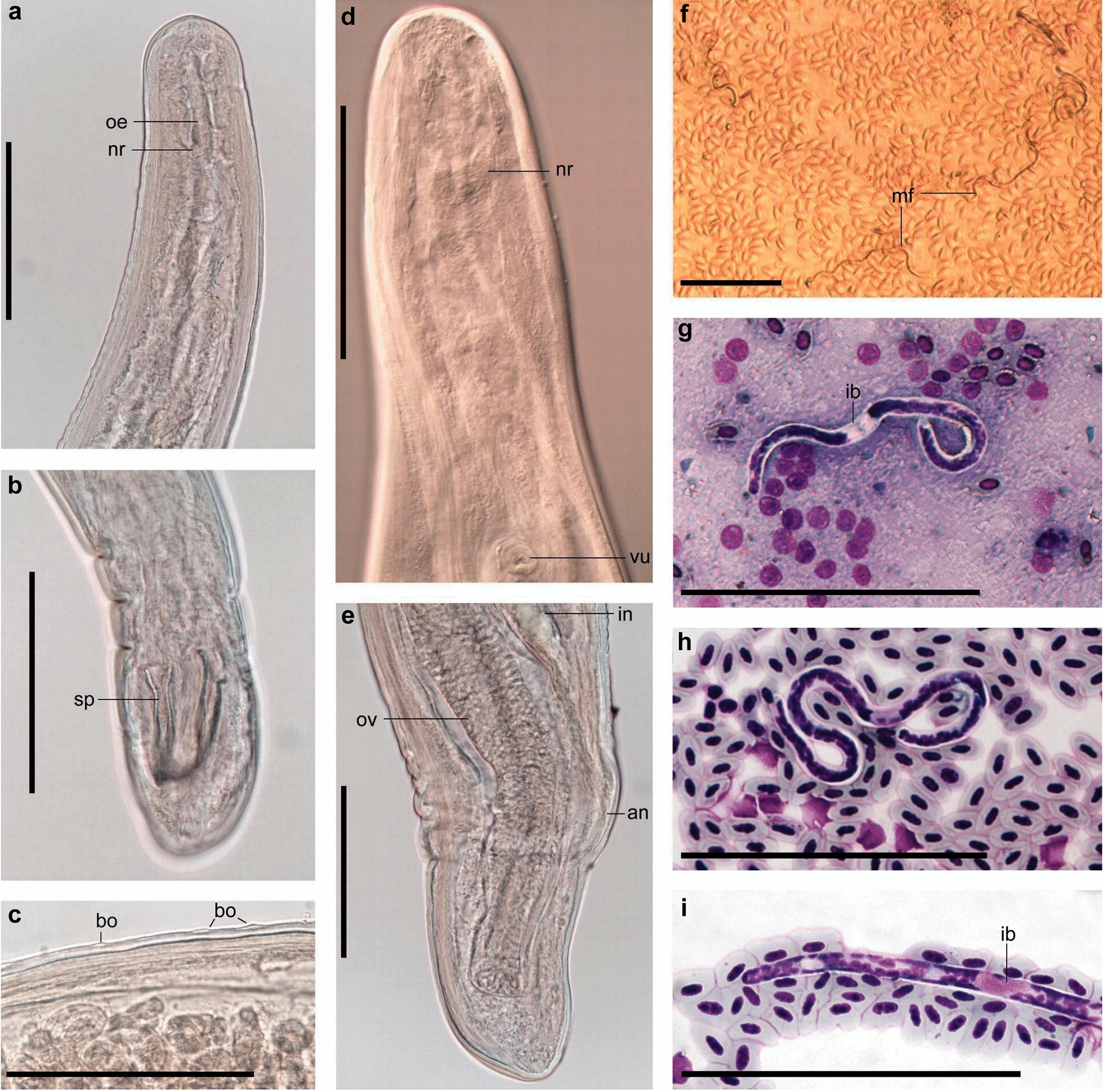
*Splendidofilaria bartletti* sp. n. from *Sylvia atricapilla*. **a** Male anterior extremity (ethanol-fixed). **b** Male posterior extremity (ethanol-fixed). **c** Cuticle with bosses (ethanol-fixed). **d** Female anterior extremity (ethanol-fixed). **e** Female posterior extremity (ethanol-fixed). **f** Live microfilaria in peripheral blood. **g** Microfilaria in liver blood film (Giemsa-stained). **h** Microfilaria in peripheral blood film (Giemsa-stained). **i** Posterior part of microfilaria from liver blood (Giemsa-stained). Note that anterior and posterior extremities of microfilariae are almost of the same width; microfilariae have tight sheath that can be seen only on blood films. Scale bars: **a**–**h** 100 μm, **i** 50 μm.* bo* bosses,* mf* microfilariae; see captions to Figs. 1 and 2 for other abbreviations

***Splendidofilaria bartletti*** Binkienė sp. n. (Figs. 2f–l, 4)

***Description:*** Slender nematodes with slightly attenuated extremities that are more easily observable in female individuals (Fig. 4a–e). Male shorter than female (Table 3). Cuticle thick, with fine transverse striations and bosses (Fig. 4c). Bosses commencing approximately 370 μm from anterior extremity in male and 434 μm in female, ending anterior to anus approximately 360 μm in male and 650 μm in female, not extended to extremities of body and not arranged in any discernible pattern. Oral opening small (Figs. 2i, 4a). Buccal capsule absent. Excretory pore at the nerve ring (Fig. 2i). Pre-esophageal ring absent. Eesophagus thin, not externally divided into muscular and glandular part. Junction of esophagus and intestines not well defined. Vulva slightly extend the body surface. Vagina short, directed posteriorly (Fig. 2i). Uterus didelphic and opisthodelphic. Ovary bends at 51 μm from posterior extremity (Fig. 4e). Spicules subequal, dissimilar, head demarcated from blade by constriction (Figs. 2j, k, 4b). Hypodermal swelling around male anus present. Two pairs of papillae are on hypodermal swelling, two papillae lateral to hypodermal swelling, two pairs postanal papillae present (Fig. 2j). Microfilariae short, with tight sheath, anterior and posterior extremity rounded (Figs. 2l, 4f–i), body width is almost the same throughout the length of the body (width at anterior extremity 3.7–4.8 μm [mean 4.3 μm]; maximum width 4.2–5.6 μm [mean 5.0 μm]; width at posterior extremity 3.5–4.5 μm [mean 4.0 μm]) (Table 2). Posterior nucleus rounded, in two lines, present at posterior extremity (Figs. 2l, 4i).

***Type host***: *Sylvia atricapilla* Linnaeus, 1758 (Passeriformes, Sylviidae).

***Location in host***: Adults in joints of toes. Microfilariae in peripheral blood, capillaries of liver and lung.

***Type locality***: Ventės Ragas Ornithological Station (55°20'28.1"N 21°11'25.3"E), Lithuanian Republic.

***Type specimens***: Holotype (female MHNG-INVE-137373) and allotype (male MHNG-INVE-137374) were deposited in MHNG.

***ZooBank accesion***: The Life Science Identifier (LSID) for *Splendidofilaria bartletti* sp. n. is urn:lsid:zoobank.org:act:D4251207-5F7E-4FB2-B728-8D121753EB30

***DNA sequences***: Mitochondrial *cox1* (GenBank accession number MT800764) and nuclear *28S* gene sequences were (MT802306) generated from microfilaria. Mitochondrial *cox1* (MT800765) and nuclear *28S* (MT802307) were also generated from adult worms.

***Etymology***: The new species is named after the Dr. Cheryl M. Bartlett, in recognition of her contribution to the knowledge of the filarioid nematodes of birds.

***Remarks***

Thirty-one species of *Splendidofilaria* have been described, most of which have been found in the blood circulatory system, mainly in the heart, aorta and other blood vessels; only three species have been found in legs: *Splendidofilaria mavis* (Leiper, 1909), *Splendidofilaria tuvensis* (Spassky and Sonin, 1957) and *Splendidofilaria bohmi* (Supperer, 1958). *Splendidofilaria bartletti* sp. n. can be readily distinguished from these three species by body, spicules and vagina length.

*Splendidofilaria mavis* was described from the song thrush *Turdus philomelos* and fieldfare *Turdus pilaris* [29]. The male of *S. mavis* is twice longer (6–11.3 μm) and wider (200–260 μm), has bigger spicules (left 76–95 μm, right 52–83 μm). The vulva opens at 400–1150 μm from the anterior extremity [29], while the vulva of *S. bartletti* sp. n. is at 267 μm from the anterior extremity. Microfilaria are slightly tapering toward the posterior extremity, while anterior and posterior extremities of microfilaria of *S. bartletti* sp. n. are of the same width.

*Splendidofilaria tuvensis* was described from the birds of the family Tetraonidae and Phasianidae [29]. Both female and males of *S. tuvensis* are almost fivefold longer (37–55 and 14–17 mm respectively). The female vagina is very long, about 1 mm, and the uterus begins at 1.5 mm from the anterior extremity, while the vagina of *S. bartletti* sp. n. is 50 μm long and the uterus begins at 387 μm from the anterior extremity. Spicules are more than twofold longer and wider (left 130–150 × 32 μm; right 110–130 × 21 μm).

*Splendidofilaria bohmi* was described from the mistle thrush [30]. The males of *S. bohmi* are longer (7–8 mm), with almost twofold longer spicules (left 102–125 μm, right 86–110 μm). Females have a longer vagina (90–190 μm). The length and shape of the microfilariae of both species are similar, but the nerve ring and excretion pore of *S. bohmi* specimens are closer to the anterior extremity. The location of the anatomical fixed points, expressed as a percentage of the total length, according to their distance from the anterior extremity is 17.9% for the nerve ring and 27.6% for the excretion pore in *S. bohmi*; the respective values in *S. bartletti* sp. n. are 19–25 and 32–41%.

### Description of microfilariae, unknown species identity

#### Microfilariae from the great reed warbler

**Fig. 5:**
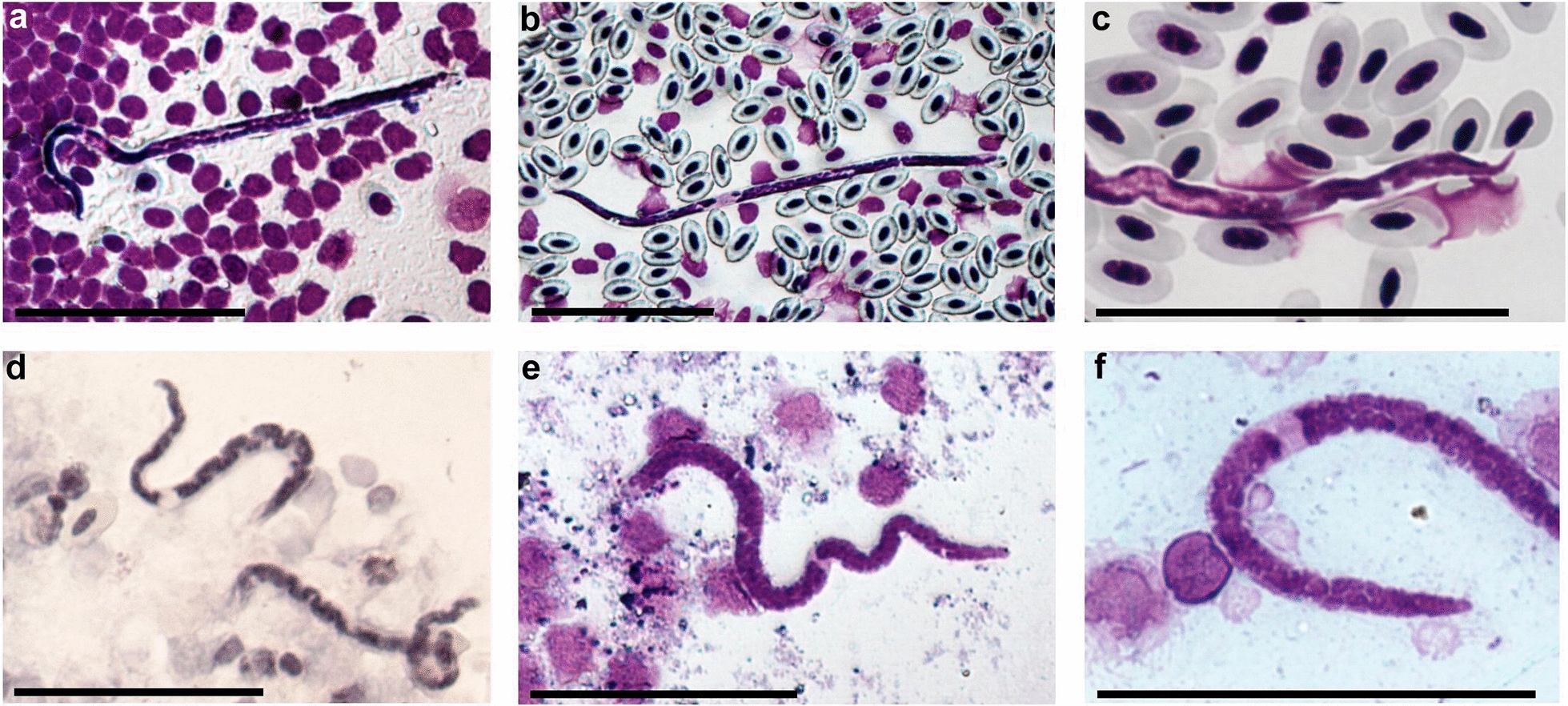
**a**–**c** Microfilariae of *Eufilaria* sp. from *Acrocephalus arundinaceus* (great reed warbler) (Giemsa-stained) thin peripheral blood films (**e**, **f**)** d** Giemsa-stained thin blood film of spleen. **d**–**f** Microfilariae of Onchocercidae sp. from *Sylvia curruca* (lesser whitethroat) Giemsa-stained thin blood films from liver. Note that posterior extremities of microfilariae are slightly tapering rounded. Scale bar: 50 μm

***Description:*** Body short (Fig. 5 a–c) with transversely striated cuticle. Anterior extremity bluntly rounded. Two minor cephalic cuticular structures present. Posterior region sharply pointed. Posteriormost nucleus rounded, present at tail extremity. Sheath absent. Measurements (with mean values in parentheses) are:

- Ten specimens from Giemsa-stained thin blood smears of lungs: length 103–154 μm (124), maximum width 3.1–4.3 μm (3.8), cephalic space 3–4.7 μm (3.8), nerve ring 25–35 μm (31), excretory pore 35–57 μm (45), excretory cell 38–60 μm (45), inner body at 58–90 μm (74) from anterior extremity, inner body length 5–10 μm (7), tale length 15–25 μm (18). The location of the anatomical fixed points, expressed as a percentage of the total length, according to their distance from the anterior extremity is as follows: cephalic space 2–4.5% (3.2%), nerve ring 21–30% (25%), excretion pore 32–43% (36%), excretory cell 37–46% (40%), inner body 56–68% (60%), anal pore 84–88% (85%).
- Two specimens from Giemsa-stained thin peripheral blood smears: length 137 μm, 118 μm; maximum width 3.5 μm, 4.6 μm; cephalic space 2.9 μm, 2.3 μm; nerve ring 29 μm, 29 μm; excretory pore 46 μm, 40 μm; excretory cell 49 μm, 43 μm; inner body at 78 μm, 64.7 μm from anterior extremity, inner body length 10 μm, 8.5 μm; tale length 22 μm, 18.2 μm. The location of the anatomical fixed points, expressed as a percentage of the total length, according to their distance from the anterior extremity is as follows: cephalic space 2.1 %, 1.9 %; nerve ring 21 %, 25%; excretion pore 34%, 35%; excretory cell 36 %, 37 %, inner body 57%, 55%, anal pore 84%, 85 %.

***DNA sequences***: Mitochondrial *cox1* (GenBank accession number MT800767) and nuclear *28S* (MT802310) generated from microfilaria.

***Remarks***

**Fig. 6:**
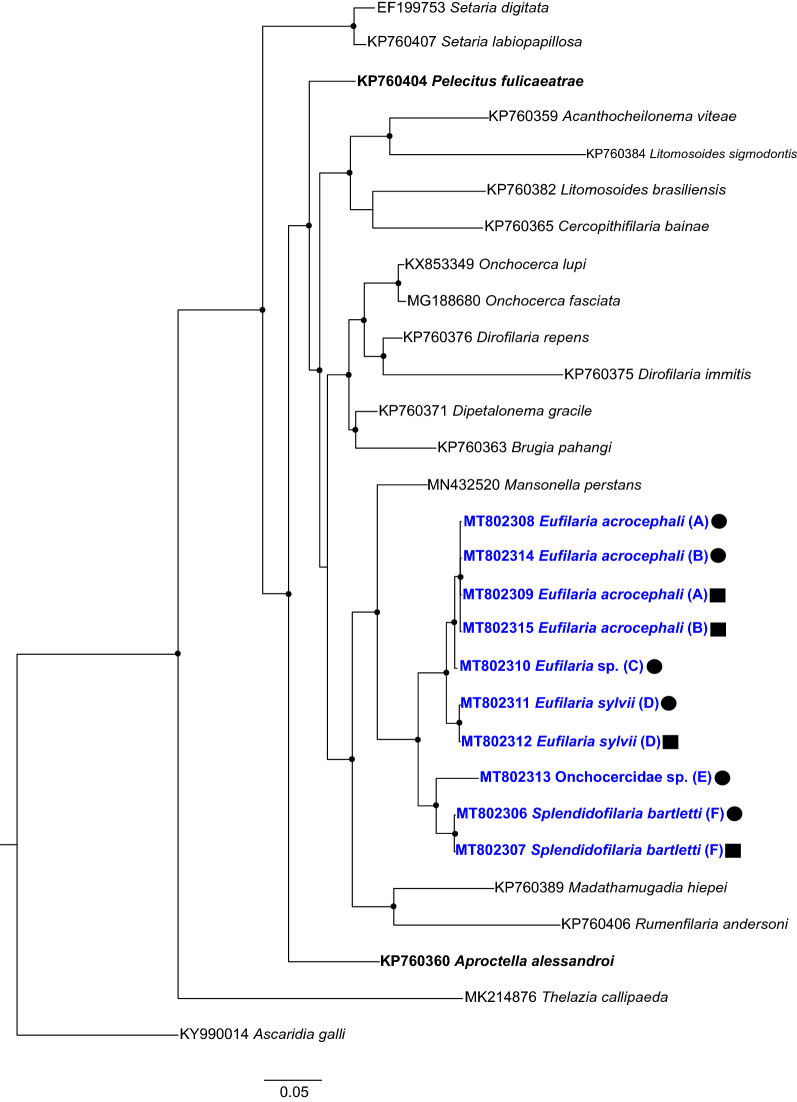
Bayesian phylogenetic inference of the nuclear *28S* gene sequences of microfilariae and adult worms found in Acrocephalidae and Sylviidae wild birds. The tree was rooted with the *Ascaridia galli* sequence. Bold font highlights filarioid nematodes parasitizing birds. Bold and blue fonts highlight sequences from this study. Black circles indicate sequences obtained from microfilariae, and black squares represent sequences obtained from adult worms. Letters in brackets indicate the host species; taxa with the same letter are those for which the sequences were obtained from the same host individual:* A*,* B* *Acrocephalus scirpaceus*,* C* *Acrocephalus arundinaceus*,* D Sylvia borin*,* E* *Sylvia curruca*,* F* *Sylvia atricapilla*. GenBank accession numbers are given. Nodes with posterior probability of ≥ 75% are indicated with dots

**Fig. 7:**
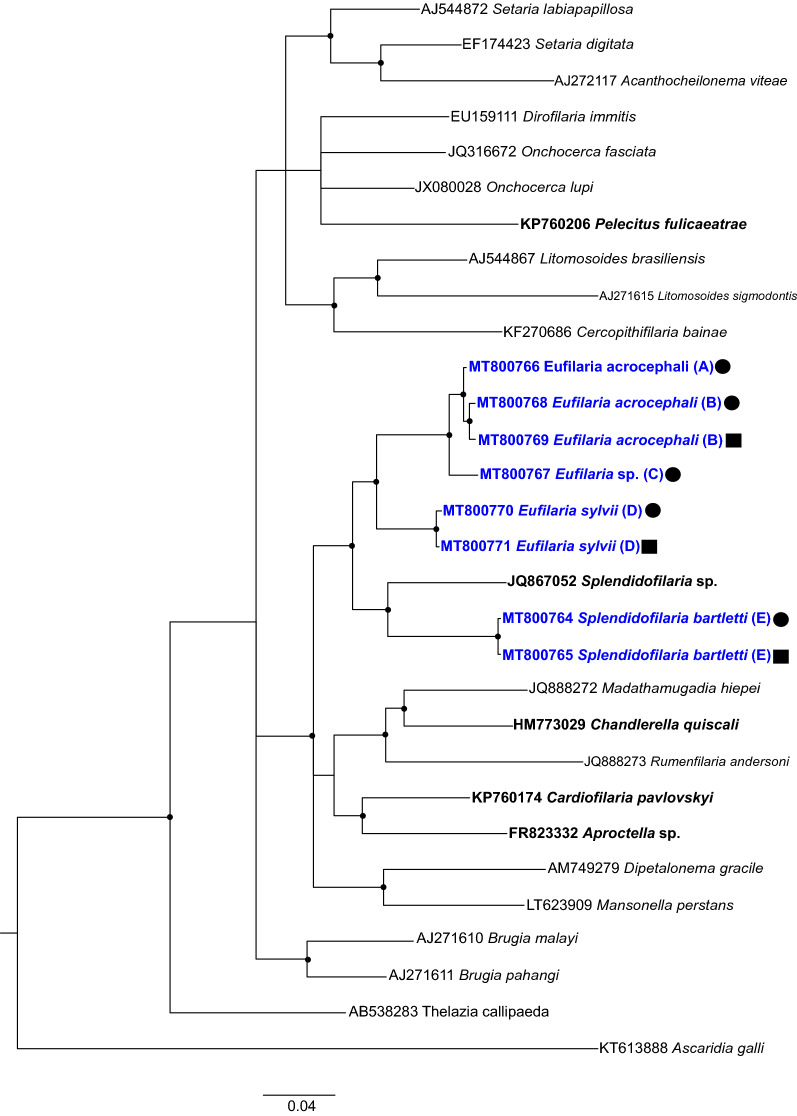
Bayesian phylogenetic inference of mitochondrial *cox1* gene sequences of microfilariae and adult worms found in Acrocephalidae and Sylviidae wild birds. The tree was rooted with *Ascaridia galli* sequence. Bold font highlights filarioid nematodes parasitizing birds. Bold and blue fonts highlight sequences from this study. Black circles indicate sequences obtained from microfilariae, and black squares represent sequences obtained from adult worms. Letters in brackets indicates the host species; taxa with the same letter are those for which sequences were obtained from the same host individual.* A*,* B* *Acrocephalus scirpaceus*,* C* *Acrocephalus arundinaceus*,* D* *Sylvia borin*,* E* *Sylvia atricapilla*. GenBank accession numbers are given. Nodes with posterior probability of ≥ 70% are indicated with dots

This parasite is similar to the descriptions of microfilariae reported in genus *Eufilaria* based on the following characters: length up to 200 μm, tail sharply pointed and sheath absent [4]. Accordingly, the phylogenetic analysis placed microfilaria from great red warbler in the same clade with other *Eufilaria* species described from warblers in the present study (Figs. 6, 7).

#### Microfilariae from lesser whitethroat

***Description:***

Ten specimens from Giemsa-stained thin blood smears of liver: body short (Fig. 5 d–f), anterior extremity bluntly rounded, slightly tapering to rounded posterior extremity. Sheath present. Posteriormost nucleus present at tail extremity. Small inner body present. Measurements (mean value in parentheses) are:

- Length 100–123 μm (112); width at anterior extremity 2.5–4.2 μm (3.2); maximum width 4.0–5.8 μm (5.0); width at posterior extremity 2.0–2.8 μm (2.4); cephalic space 1.3–2.5 μm (1.8); nerve ring 22–27 μm (24); excretory pore 32–44 μm (38); excretory cell 35–49 μm (42), inner body at 60–69 μm (64) from anterior extremity, inner body length 1.3–3.4 μm (2.2); tale length 12–15 μm (13). The location of the anatomical fixed points, expressed as a percentage of the total length, according to their distance from the anterior extremity is as follows: cephalic space 1.2–2.2% (1.6%), nerve ring 22–27% (24%), excretion pore 30–37% (33%), excretory cell 33–41% (37%), inner body 56–61% (58%), annal pore 86–90% (88%).

***DNA sequences***: Nuclear *28S* (MT802313) gene sequence was generated from microfilaria.

***Remarks***

This parasite corresponds to the description of microfilariae reported for the genus *Paronchocerca*: length up to 200, tail tapering rounded, sheath present [4]. Because no adult nematode was found, we cannot confirm that this microfilaria belongs to the nematodes of the genus *Paronchocerca*; therefore in the phylogenetic tree it is named as Onchocercidae sp. (Fig. 6). The phylogenetic analysis placed the microfilaria from lesser whitethroat close to the new species of *Splendidofilaria* described in the present study (Fig. 6, 7).

### Sequence data and phylogenetic analysis

In all, 18 new sequences were obtained (8 of the *cox1* gene and 10 of the*28S* gene). We did not manage to obtain *cox1* gene sequences of adult worms detected in one *A. scirpaceus* individual and of microfilaria detected in *S. curruca.*

Phylogenetic analysis using both genes placed DNA sequences of parasites of different genera in different well-supported closely related clades. Both phylogenies were of similar topology (Figs. 6, 7). In both trees, DNA sequences of the closely related *Eufilaria* species appeared in one well-supported clade (Figs. 6, 7), indicating their close phylogenetic relationship. Both phylogenies readily separated reported parasites of the genera *Eufilaria* and *Splendidofilaria*, indicating that phylogenies based on both genes can be used for predicting phylogenetic and taxonomic position of as yet non-identified parasites to species levels. The separate position of DNA sequences of the parasites belonging to different genera in the trees (Figs. 6, 7) supports the validity of these genera.

## Discussion

Blood parasites are markedly diverse and have been the subject of much research, not only by helminthologists but also by protistologists, including the area of avian malaria research, resulting in a large number of publications reporting the presence of microfilariae in the circulatory system [1–3, 14, 16, 18, 43–48]. However, little progress has been made in the study of blood parasite phylogenies, microfilaria taxonomy and biology in wildlife due to difficulties in species identification. Here, we report the identification of 18 new barcode DNA sequences of avian filarioid nematodes. The key results of this study are: (i) the description of three new species and their molecular characterization using *28S* and *cox1* gene partial sequences; (ii) molecular proof of the taxonomic value of morphological characters of circulating microfilariae in the identification of species and genera of avian filarioids; (iii) the molecular support of the validity of the genera *Eufilaria* and *Splendidofilaria*; and (iv) a suggestion that the *28S* gene sequences are appropriate tools for species identification of avian Onchocercidae. These points were discussed in more detail below.

### Species taxonomy and the role of microfilariae in the identification of avian filarioids

Our study revealed that species diversity of avian filarioids remains insufficiently investigated. Interestingly, each positive bird species was infected with readily distinguishable parasite species in this study, indicating the existence of markedly underestimated parasite diversity, which might exceed bird species diversity and certainly worth further investigation. Importantly, the new species and their genera can be readily identified using only morphological characters of circulating microfilariae. This provides opportunities to predict parasite taxonomic position based on visualization of these microfilariae, which are available in blood film collections in many museums. In other words, a large and as yet unexplored reserve of microfilariae studies is available for future research because microfilariae are present in blood film collections in many prominent museums and can be used for such investigations. These parasite depositaries are the Collection of International Reference Centre for Avian Haematozoa (Queensland Museum, Australia); American Museum of Natural History (USA); Natural History Museum (London, UK); University of Nebraska State Museum (USA) [1, 2, 14, 49], and all have provided valuable reviews on reports of microfilaria in birds that have not be identified to the species level. Further studies and more information on microfilaria morphology and molecular characterization of filarioid nematodes are needed for the identification of possible patterns in the diversity and distribution of these parasites.

In this study, filarioid nematodes were found in birds of the families Acrocephalidae and Sylviidae for the first time. The examined birds breed in Europe and migrate to Africa during the cold period of the year [50]. Experimental studies of the life cycles of *Splendidofilaria fallisensis* (Anderson, 1954), *Splendidofilaria picacardina* Hibler, 1964 and *Eufilaria longicaudata* Hibler, 1964 show that microfilariae appear in the blood 30–76 days after infection [5]. It is therefore most likely that the examined birds of the families Acrocephalidae and Sylviidae were infected with filarioid nematodes outside of the breeding grounds. An investigation of juvenile birds that hatched in Europe is needed to prove if transmission of the new species also occurs at European breeding grounds.

Due to lack of molecular characterization of the majority of described filarioid species, examination of adult worms in parallel with their microfilariae currently is essential for species identification. The adult worms are challenging to collect because of their internal host localization and ephemerality of some species. Additionally, even if the adults are found, their species identification is often difficult due to simple external morphology, which requires good taxonomic knowledge of morphological features of this group [4]. These are the prominent obstacles for the diversity research. It is easier to detect circulating microfilariae, particularly because euthanasia of the host is unnecessary [20]. This study combined traditional morphological characters with molecular markers, and the results support previous speculations that identification of circulating microfilariae is possible at both the genus and species levels in wild birds [4]. However, it remains unclear how broadly microfilariae morphology can be applied for parasite species identification. Further studies and accumulation of additional information is needed to specify this issue, which is important to speed filarioid parasite biodiversity research in wildlife.

The results of this study show that there is a relatively high degree of variability in some morphological and morphometric characters of microfilariae—even in the same species of parasite—that are present in the same avian host. For example, the microfilariae of *S. bartletti* sp. n. were shorter in blood films prepared from blood from the liver than in blood films prepared from the peripheral blood (Table 2). Accordingly, the distance from the anterior extremity to the inner structures of microfilariae were shorter, but the relative distances of fixed points expressed as a percentage of total length were similar. The length of microfilariae varies not only in blood taken from different organs, it may also vary depending on the fixation procedure used. Microfilariae of *Eufilaria delicata* fixed in cold are shorter than those fixed in hot ethanol [41]. There are also minor differences in the length of microfilariae from fresh and frozen material [51]. Therefore, the methods for blood film preparation and collection of morphometric data to be used in keys of microfilaria identification must be standardized for the detection of characters. However, some morphological characters of microfilariae are relatively stable independently of blood film preparation and can be preferably used for parasite identification. Among these characters are the shape of the anterior and posterior extremities, the shape and location of nuclei and the presence or absence of the sheath; it is these characters that should be examined first the parasites to be examined. It is often impossible to take measurements and to examine the morphology of live microfilariae located in organs during fieldwork. However, microfilariae in the periphereal blood can be readily observed in fixed stained peripheral blood preparations, and these parasites have often been reported in studies addressing other blood parasites [14, 18, 43–47, 52–57].

Blood films can be used for the morphological identification of microfilariae. We suggest preparing at least five blood films from parasite-positive birds because parasitemia intensity may be low, and the number of microfilariae in the blood may not be sufficiently high for measurements and precise examinations. Importantly, the preparations should be rapidly air-dried, fixed by immersing in absolute methanol and stained with Giemsa solution (for these methods see [21]). It is advisable that the morphometric description of microfilariae should include distances of fixed points (cephalic space, nerve ring, excretory pore, excretory cell, inner body, anal pore) expressed in micrometers and as a percentage of total length (see Table 2). These are particularly valuable taxonomic characters.

The main known morphological features of microfilariae of avian filarioids of different genera were summarized by Bartlett [4]. Unfortunately, there is no information on the morphology of microfilariae of the genera *Pseudlemdana*, *Striatofilaria* and *Aproctiana*. This study showed that microfilariae of some species of *Splendidofilaria* (*S. algonquinensis* (Anderson, 1955) Anderson, 1961, *S. wehri* Anderson, 1961 and *S. bartletti* sp. n.) have a tight sheath, while microfilariae of other species of this genus are unsheathed [26, 58, 59]. Therefore, our results demonstrate that the keys of microfilariae of avian filarioids [4] should be supplemented with information on the presence of the tight sheath in parasites of the genus *Splendidofilaria.*

Even though morphological identification of microfilariae is complicated due to the simple morphology and the similarity of different species, many microfilariae have readily distinguishable specific characters, which can be used during identification. For example, the microfilaria of *E. acrocephalusi* sp. n. can be distinguished from other described microfilariae of this genus by its very thin and long body and absence or presence of very short (up to 1.8) cephalic space (Fig. 1e–h). Some other microfilariae of this genus also have specific characters. For example, *Eufilaria coua* (= *E. singhi* Chabaut, Brygoo, Richard, 1964) Anderson, Prestwood, 1969 are short and thick (65 × 6 µm); the inner body of *Eufilariella delicata* Supperer, 1958 is long (16–17 µm); the inner body of *Eufilaria mcintoshi* Anderson and Bannett, 1960 is situated in the last third part of the body (66–74 % from anterior extremity) while in other *Eufilaria* spp. it is located behind the middle line of the body (51–58%) [29, 30, 60]. The microfilaria of *E. sylviae* sp. n. can be distinguished from those of *E. acrocephalusi* sp. n. due to the former’s sharply pointed curved tail (compare Fig. 3h with Fig. 1i), wider body, longer cephalic space and absence of nuclei at the posterior extremity (Table 2). Information on the morphology of most avian microfilariae is absent or limited; therefore, further studies are needed to collect information on this subject.

### Recent advances in molecular characterization of avian filarioid nematodes

The application of molecular diagnostic tools has highlighted the diversity of filarioids and is essential for the identification of species, developing classification systems and DNA barcoding [61]. Currently, only a few studies have looked at adult worms of avian Onchocercidae parasites. The adult worms studied are: *Chandlerella quiscali* and *Splendidofilaria* sp. (*18S* rDNA and *cox1* genes sequences were used [11]); *Eulimdana clava* (*cox1* and *12S* rDNA [12]); and *Cardiofilaria pavlovskyi*, *Aproctella alessandroi* and *Pelecitus fulicaeatrae* (*12S* rDNA, *cox1*, *rbp1*, *hsp70*, *myoHC*, *18S* rDNA and *28S* rDNA [13]). Several studies have also reported DNA sequences, which were recovered from unidentified microfilariae in the peripheral blood of birds [14, 34]. Additionally, several studies have reported DNA sequences of unknown species of filarioid nematodes in mosquitoes [15–17, 62, 63]. Due to scarcity of available sequence information, generalizations on parasite molecular biology would be premature at this time.

In this study, we used the *cox1* gene in our analysis because it is the most commonly used gene in animal barcoding [9, 64] and the nuclear *28S* gene for parasite species identification. Even though phylogenetic trees based on these genes had a similar topology (Figs. 6, 7), the DNA sequences detected from the same parasite species were more diverse in *cox1* than in *28S*, illustrating that the use of *28S* gene is preferable in taxonomic studies with avian Filarioidea species.

The results of the phylogenetic analysis in our study support current morphological observations that the birds of the genus *Acrocephalus* are infected at least with two species of nematodes of the genus *Eufilaria* (Figs. 6, 7). Microfilariae of these parasites were readily distinguishable morphologically (compare Figs. 1e–h and 5d–f). Unfortunately, adult nematodes in the great reed warbler were not found in microfilariae-positive hosts, and it was impossible to compare the morphology of this parasite at the adult nematode stage with the parasite found in common red warblers. However, the morphological comparison of microfilariae revealed some differences between these phylogenetically closely related species. Microfilariae from the peripheral blood of the great red warbler are wider; possess a longer inner body and a bigger cephalic space; and nuclei are not arranged in two rows (Fig. 5d-f); these observations suggest that these bird species are infected with different microfilariae species.

The phylogenetic analysis using *28S* gene sequences revealed that all studied microfilariae species found in *Sylvia* birds belongs to different species (Fig. 6). The adult nematodes were not found in the lesser whitethroat, but morphological examination of the microfiliariae showed that this bird most likely was infected with parasites of another genus of subfamily Splendidofilariinae, the genus *Paronchocerca*. Further studies of adult worms are needed to prove this assumption.

The phylogenetic analysis of *cox1* and *28S* gene sequences indicates that each species of bird harbors different species of filarioids (Figs. 6, 7). In addition, all studied species appeared in one well-supported clade, indicating their closed phylogenetic relationships.

## Conclusions

Three new species of avian filarioid nematodes are described and their molecular characterization defined. The limited data currently available suggest that species diversity of avian filarioids is markedly underestimated and that the number of species may be higher than the number of bird species. This finding calls for further biodiversity studies of this group of helminths. Our molecular data showed that the morphological characters of adult worms, which have been traditionally used in the taxonomy of Filarioidea species, do have a phylogenetic value. Importantly, the parasites of different genera can be distinguished by the morphology of their microfilariae. This calls for further precise research on morphology of avian microfilariae. DNA sequences of the *28S* gene were particularly informative in terms of identifying these parasite species. The linkage of molecular and morphological approaches is deserving of more attention in Filarioidea species research, particularly because this approach provides new knowledge that will improve our understanding of these insufficiently investigated pathogens without causing much harm to wildlife.

## Supplementary Information

**Additional file 1: Table S1.** Pairwise distance (p-distance) was calculated between sequences used in the phylogenetic analysis for 28S gene. This analysis was implemented in MEGAX software using the p-distance model with uniform rates of nucleotide substitutions.

**Additional file 2: Table S2.** Pairwise distance (p-distance) was calculated between sequences used in the phylogenetic analysis for *cox1* gene. This analysis was implemented in MEGAX software using p-distance model with uniform rates of nucleotide substitutions.

## Data Availability

All data generated or analysed during this study are included in this published article. All newly generated sequences were submitted to the GenBank database. The type and voucher material (see parasite descriptions) was deposited in the Natural History Museum of Geneva and in the Nature Research Centre, Lithuania.

## Abbreviations

*cox1*: Mitochondrial cytochrome* c* oxidase I
MHNG: Natural History Museum of Geneva
NRCL: Nature Research Centre, Lithuania
LSID: The Life Science Identifier
